# Live Hospice Discharge of Individuals with Cognitive Disabilities: A Systematic Review

**DOI:** 10.1016/j.jamda.2025.105578

**Published:** 2025-04-09

**Authors:** Victoria M. Winogora, Christine E. DeForge, Kimberlee Grier, Patricia W. Stone

**Affiliations:** 1 Columbia University School of Nursing, New York, NY, USA

**Keywords:** Hospice and end-of-life care, Cognitive Disability, Dementia, Adult and older adult

## Abstract

**Objectives:**

To systematically review the evidence on live hospice discharge for individuals with cognitive disabilities.

**Design:**

Systematic Review

**Setting and Participants:**

Adults with cognitive disabilities enrolled in hospice in the United States

**Methods:**

Following the Preferred Reporting Items for Systematic Reviews and Meta-Analyses (PRISMA) guidelines, we searched for United States-based (US), English-language, and peer-reviewed literature focused on live discharges from hospice for individuals with cognitive disabilities. We searched PubMed, CINHAL, and Web of Science for articles published between January 1, 2014, through August 1, 2024. We used the Joanna Briggs Institute Analytical Cross-Sectional Studies Appraisal Tool to assess study quality.

**Results:**

After screening 1,543 titles and abstracts, we completed a full text review of 30 articles, of which 8 met inclusion criteria. All included studies were cross-sectional analyses. The indications of cognitive disability varied (i.e., dementia diagnosis, positive result on cognitive function assessment), but there were no studies focused on individuals with acquired brain injuries or intellectual and developmental disabilities, nor was the term cognitive disability used in any of the studies. In all studies, the indicator of cognitive disability was associated with live hospice discharge. Other risk factors included female sex (n =4), minoritized race (n=4), forprofit hospice ownership (n = 4) and delivery of hospice services at home (n=2). In all studies, researchers found that individuals with cognitive disabilities had longer hospice lengths of stay.

**Conclusions and Implications:**

This systematic review is the first to focus on live discharge from hospice for individuals with cognitive disabilities. All studies focused exclusively on individuals with dementias. While the term cognitive disability was absent from the literature reviewed, cognitive disability was associated with live discharge. Future research should aim to include the greater cognitive disability community to assess hospice and other end-of-life outcomes to identify potential targets for future intervention.

## Summary of article

This review examines live hospice discharge of people cognitive disabilities. In all included studies cognitive disability was associated with live hospice discharge, indicating disparities in hospice quality for this population.

## Introduction

Cognitive disabilities are defined as serious difficulty concentrating, learning new things, remembering, or making decisions.^1,2^ These disabilities are commonly associated with several diagnoses (i.e., Alzheimer’s disease and related dementias [ADRDs], stroke, traumatic brain injuries, intellectual and developmental disabilities).^1^ Except in the context of ADRDs, individuals with cognitive disabilities are commonly underrepresented in end-of-life research for a variety of reasons.^2–4^ Even among individuals with ADRDs, under-detection and/or late diagnosis can contribute to their underrepresentation.^5^ For example, it has been estimated that 41% of Medicare decedents have dementia, yet using data from 2022, the Centers for Medicare and Medicaid Services reported only 11.1% having an official diagnosis of ADRDs.^3,6,7^ This difference may be because nearly half of patients with ADRDs are not diagnosed until the later stages, suggesting that use of ADRDs diagnostic codes in research may result in unintentionally excluding a large segment of the population with cognitive disabilities.^5,8^ Other contributing factors, including ethical considerations and medical mistrust, influence research participation for other individuals with cognitive disabilities, including those resulting from intellectual and developmental disabilities.^9^ Using the term cognitive disability in research better captures this population, who are vulnerable to poor/sub-optimal end-of-life outcomes.^10–12^

One and a half million Medicare beneficiaries annually use hospice care, a specialized service available to individuals near end-of-life (i.e., with a terminal condition and < 6-month prognosis.,^13–15^ Data describing hospice use among individuals with cognitive disabilities broadly speaking is lacking; much more is known about hospice use among those with ADRDs. Roughly 20% of individuals who enroll in hospice are diagnosed with ADRDs.^14,15^ Researchers have found that individuals with ADRDs are less likely to enroll in hospice in the last month of life compared to individuals without ADRDs.^16^ Little is known about end-of-life care for individuals with intellectual and developmental disabilities; however data from small qualitative studies demonstrate that these populations are accessing palliative and end-of-life care.^10–12,17^ Given that hospice is considered best practice for end-of-life care, because it minimizes unwanted treatments and improves quality of life near end-of-life, it is essential to understand barriers to high-quality hospice care for this vulnerable population.^18–20^

Disparities in hospice access and care quality (e.g., length of stay) for individuals with cognitive disabilities have been identified in recent literature.^11,12,21,22^ Concurrently, a troubling trend of increased live discharge from hospice for individuals with ADRDs has emerged.^13,14,23,24^ Live hospice discharge is defined as termination of hospice benefits and discharge of a patient from hospice services prior to the patient’s death, that is, while the patient is alive.^13,14,23,24^ Data from the National Home and Hospice Care Survey found that between 6% and 15% of hospice patients experience live discharge.^25^ Live discharge from hospice can happen for a variety of reasons (e.g., patient/family preferences to pursue life-prolonging treatment and/or hospital admissions); however, the concern is that, for individuals with cognitive disabilities, live discharge often happens when a patient has outlived their hospice prognosis (i.e., > 6 months) and therefore the benefit is terminated. Roughly 20% of all hospice patients are discharged alive, and comorbid dementia diagnosis places an individual at increased odds of live discharge.^7,14,23,26^ Qualitative researchers have described the detrimental impacts of live discharge on patients and families, resulting in poor end-of-life care quality and suboptimal bereavement outcomes.^13,14,27,28^

Because individuals with cognitive disability are at risk for sub-optimal end-of-life care,^12,29,30^ an understanding of current hospice care practices among this group, specifically live discharge and factors influencing live discharge, is essential. Thus, the purpose of this systematic review is to synthesize the evidence on live hospice discharge for individuals with cognitive disabilities, including factors influencing differences in live hospice discharge (e.g., patient characteristics and hospice agency characteristics).

## Methods

We developed and registered an *a priori* protocol (PROSPERO CRD42024571021) and followed the Preferred Reporting Items for Systematic Reviews and Meta-Analyses (PRISMA) guidelines for the conduct and reporting of this review. A comprehensive search strategy was developed (Supplemental Methods) in consultation with a research librarian and used to search the following databases from January 1, 2014, through August 1, 2024: PubMed, CINAHL, and Web of Science. We excluded studies published prior to 2014 due to the Improving Medicare Post-Acute Care Transformation (IMPACT) Act of 2014, which systematized audits of hospices with high proportion of long stays, directly impacting the federal regulations surrounding hospice care.^31^ Studies meeting inclusion criteria: 1) included both observational and interventional studies; 2) were conducted in the US to limit differences across geographic and health care system contexts (e.g., health care and cultural variations to end-of-life care); 3) were published in peer-reviewed journals in English; 4) included adult (>18 years of age) patients enrolled in hospice care; 5) included patients with a cognitive disability (i.e., diagnosis of dementia, stroke, brain injuries, intellectual or developmental disability or evaluated to have cognitive impairment at the time of hospice care delivery); and 6) examined live discharge from hospice care. We excluded studies that 1) were qualitative, due to the nature of the review question; 2) included in-hospital (general inpatient) hospice due to Medicare requirement that these encounters are short-term; 3) were written in a language other than English; 4) were conducted outside the US; 5) did not include individuals with cognitive disabilities; 6) did not assess hospice care; and 7) did not assess live discharge from hospice care.

### Article Screening

All identified records were uploaded into Covidence^32^, which was used for duplicate removal and to manage review workflow. All team members reviewed a subset (n =15) of titles/abstracts together to establish consistency between reviewers. Two reviewers (VMW, PWS) then independently screened the remaining titles/abstracts for eligibility and full texts of potentially eligible records. Discrepancies were resolved through team discussion.

### Critical Appraisal of Study Quality

As only cross-sectional studies were included in this review the Joanna Briggs Institute Analytical Cross-Sectional Studies Tool (see Supplemental Materials) was used to assess study quality; each quality criterion was categorized as Yes, No, Unclear or Not Applicable.^33^ Each item response coded as “yes” was scored as one point and “no,” “unclear,” and “not applicable” was scored as zero, yielding a total score, with higher scores indicating higher quality. To standardize assessments across studies, overall quality was calculated as the proportion of “yes” responses to the total number of items in the study designs checklist. We deemed studies earning < 50% of Joanna Briggs Institute elements to be at high risk of bias (i.e., low quality). Two reviewers independently assessed study quality. Discrepancies in quality assessments were discussed by reviewers and, additional team members were enlisted if needed to reach consensus. If low quality studies were found, we excluded them from data synthesis.

### Data Extraction

Two independent reviewers extracted data from each included study, using a standardized data extraction form, which was pilot tested prior to use. We extracted the following data: article citation, study aims, study outcomes, inclusion criteria including how cognitive disability was identified, exclusion criteria, data source, sample size, dates of data collection, study location, statistical analysis method utilized, covariates, patient demographics (e.g., race, sex, age, socioeconomic status, primary hospice diagnosis, and comorbidities), hospice level data (e.g., geographic location, urbanicity, and ownership), and hospice quality data (e.g., nurse visits and hospice quality rating). Discrepancies in extracted data were resolved through team discussion.

### Data Synthesis

Study data were synthesized using tables to facilitate comparisons. We grouped studies by participant diagnoses, sex, setting, and location. Tables were created to facilitate comparisons across studies. This method was selected to allow for comprehensiveness. As consistencies began to appear across studies, major findings were put onto tables for ease of perception.

## Results

**Figure 1.**
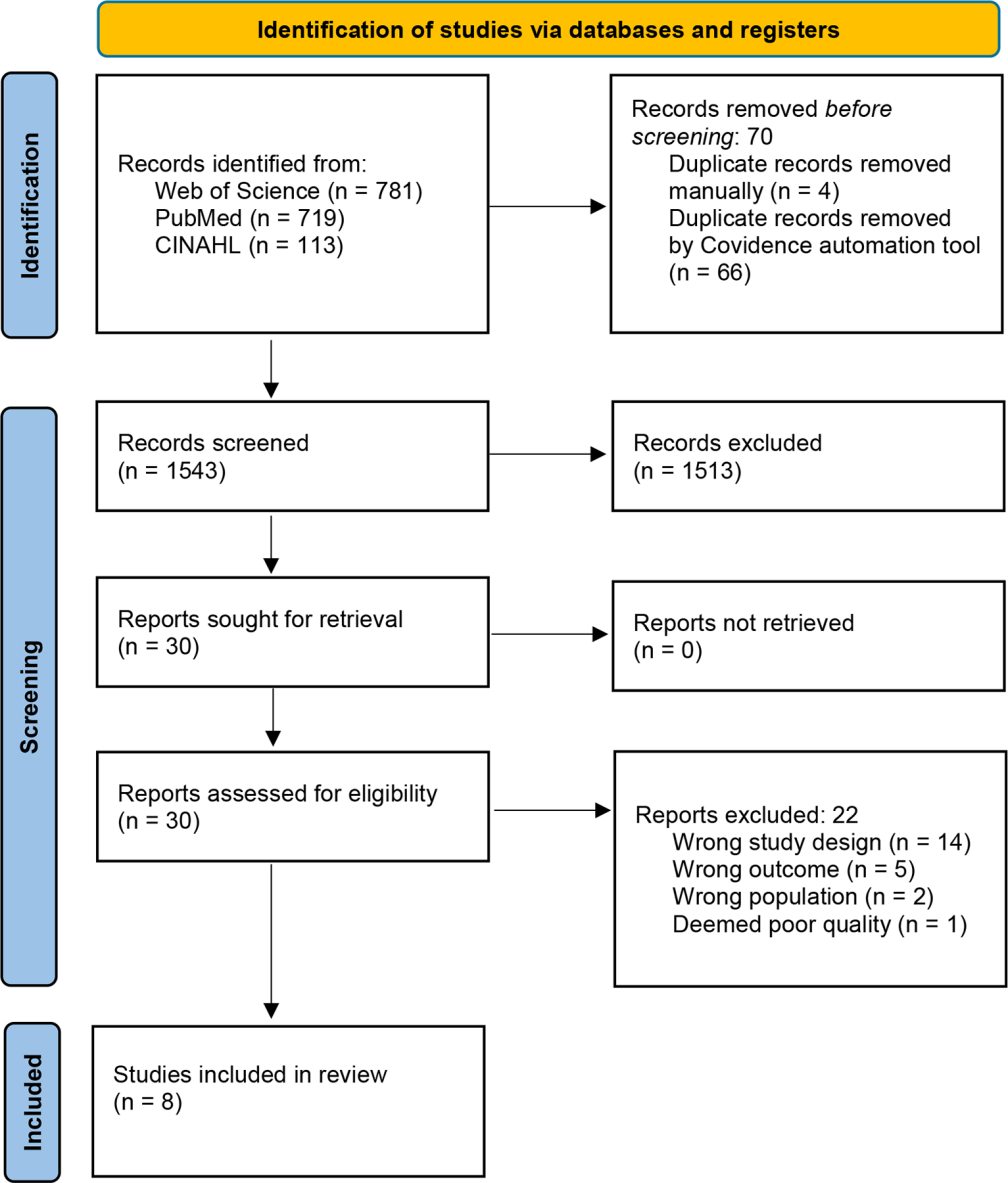
PRISMA 2020 flow diagram for new systematic reviews which included searches of databases and registers only *Consider, if feasible to do so, reporting the number of records identified from each database or register searched (rather than the total number across all databases/registers). **If automation tools were used, indicate how many records were excluded by a human and how many were excluded by automation tools.

The initial search yielded 1,543 records after removing duplicates; 30 full article texts were assessed for eligibility and 8 studies ultimately met criteria for inclusion (Figure 1). A complete list of excluded studies with justification is provided in the Supplement/Appendix.

### Quality Appraisal

Table 1 in the Supplemental Materials shows the results from the quality appraisal conducted for this review. Six of the eight studies included in this review scored 100% on Joanna Briggs Institute appraisal.^13,14,22,24,34,35^ Two of the studies^7,23^ were assessed as unclear in two elements: 1) was the exposure measured in a valid and reliable were way? and 2) were objective, standard criteria used for measurement of the condition? Both studies^7,23^ were scored at an 80%, and upon discussion were determined to be suitable for inclusion.

### Study Characteristics

Table 1 presents the characteristics of the included studies. All the studies involved secondary data analyses. The studies were published in 2017,^34^ 2018,^22^ 2020,^23^ 2021,^24^ 2022,^7,14^ and 2023.^13,35^ There were variations in location and types of hospices included in the analyses. In relation to geographical variation, authors of 3 studies looked at single hospice providers in New York City (NYC),^23,24,34^ whereas 5 looked at national hospice data.^7,13,14,22,35^ Samples ranged in size from 2,629^23^ to 2,195,076.^13^ The drastic difference in range is explained by three of studies with smaller sample sizes that used data from single hospice agencies in NYC,^23,24,34^ and one group that used data from the Health and Retirement Study.^7^ In the studies with larger sample sizes, researchers used data from national data sources.^13,14,22,35^ In three studies, investigators calculated mean age,^7,23,24^ which ranged from 82.8^7^ to 89.8 years of age.^23^ For all the studies included in this review, investigators included sex and race; and in every study, females outnumbered males, with the range being 58%^34^ to 72.2%^24^ female. Race was broken down into categories. Black non-Hispanic subjects ranged from 6.7%^14^ to 16.1%^32^ of total participants. Hispanic subjects ranged from 1.7%^13^ to 21%^24^ of total participants. In 6 studies, researchers focused on patients with dementia,^7,14,22–24,35^ whereas 2 looked at live discharges more generally, and conducted subset analyses on individuals with dementia.^13,34^

### Measurement of Cognitive Disability

In five studies, investigators used ICD-9/ICD-10 codes to determine cognitive disabilities (i.e. ADRDs).^13,14,22,34,35^ In another study, the investigators also used ICD-9/ICD-10 codes for patients with primary diagnoses of ADRDs, and a probability score of > 0.5 for patients they determined had comorbid dementia.^7^ In two studies investigators used electronic medical record data (i.e., cognitive assessments) to determine if a patient had dementia.^23,24^

Six studies looked at individuals with primary hospice diagnoses of dementia versus individuals with other primary hospice diagnoses.^7,22–24,34^ Russell and peers (2017) compared individuals with a primary hospice diagnosis of dementia versus those with cancer, heart failure, stroke, and pulmonary disease.^34^ Lastly, Gianattasio and peers (2023) compared individuals with a primary hospice diagnosis of dementia to those with lung cancer.^13^

### Other Factors Assessed for Association with Live Discharge

In four studies researchers assessed the relationship between race and ethnic minority status and live hospice discharge.^7,14,34,35^ Sex was assessed in four of the included studies.^7,14,22,34^ Location of hospice delivery was assessed in two of the included studies.^23,24^ In four studies, investigators examined the for-profit status of the involved hospice agencies.^13,14,22,35^ Lastly, in two studies researchers described the impact of registered nurse visits on live hospice discharge.^23,24^

### Study Findings

Following death, live discharge is the second most common outcome for individuals with cognitive disabilities receiving hospice care.^14,22,24,24,34^ In two studies, researchers found that live hospice discharge occurred in the context of condition stabilization, acute hospitalization, transfer or service move, and elective revocation.^23,34^ In six studies, researchers found that individuals with ADRDs experienced live discharge at increased rates compared to individuals with alternative primary hospice diagnoses, such as cancers, heart failure, stoke, and pulmonary disease.^7,13,22–24,34^ In these six studies, the authors found that the percentage of live discharge for individuals with cognitive disabilities ranged from 21%^24^ to 45%.^7^ In all (n=8) studies, researchers found that individuals with indicators of cognitive disabilities had longer lengths of stay in hospice. Authors of all studies included in this review (n = 8) reported that prolonged length of stay is associated with live discharge in individuals with an indicator of cognitive disabilities. Two additional elements were of particular interest:

1. **Patient-Level Factors Other than Cognitive Disability Were Associated with Live Discharge.** Table 2 presents factors associated with live discharge across studies. Demographic factors other than cognitive disability that were associated with live discharge included minoritized race or ethnicity^7,14,34,35^ and female sex.^7,14,22,34^ Further, minoritized race was found to be a risk factor for live discharge (both in national^14,35^ and NYC samples)^23,24^ in addition to female sex (also both in a NYC sample^34^ and national samples).^7,14,22^
2. **Hospice-Level Factors Were Associated with Live Discharge**. Hospices in the lower quartile of quality ratings (as measured by the Consumer Assessment of Healthcare Providers and Systems survey for hospice), and those that were for-profit hospices were identified as risk factors for live hospice discharge for patients.^13,14,22,35^ Hunt and colleagues (2022) found no significant regional variation in relation to live hospice discharge for patients with dementia.^14^ Other hospice level factors identified with live discharge included hospice care delivery in the home setting,^23,24^ and greater time between registered nurse visits.^23,24^ Specifically, in two single-agency (NYC; non-profit) studies, researchers found that live discharge risk increased as the number of days between nurse visits increased.^23,24^

## Discussion

To our knowledge, this is the first systematic review assessing live hospice discharge among individuals with cognitive disabilities. The authors of the research synthesized did not use the term “cognitive disability.” Nevertheless, in all the research synthesized we found that the indicator of cognitive disability was associated with increased likelihood of live discharge compared to those with other diagnoses (i.e., cancer, pulmonary diseases, heart failure), which warrants further exploration.^7,13,34^ We identified patient-level risk factors for live discharge (i.e., female sex, racial and ethnic minority, and prolonged length of stay).^14,22,23,34,35^ Furthermore, we found that live discharge was associated with hospice ownership, location of hospice care delivery, and geographic location.^13,22–24,35^

Other researchers have conducted systematic reviews of live discharges from hospice specifically in ADRDs patients.^15^ We expanded upon this work by synthesizing findings relating to both patient-level (e.g., diagnosis, location of care, race/ethnicity, sex) and organizational factors (e.g. ownership, geographic location, quality) associated with live discharge from hospice and including diagnoses other than ADRDs.

In a qualitative study, researchers found that live discharge is distressing to patients and families and results in suboptimal hospice outcomes (i.e. poor symptom control, acute care hospitalizations).^28^ Identifying the patient level factors associated with live discharge provides direction for future research, both to explore reasons behind these differences and also to develop interventions to avoid live discharge for these specific groups.

In the included studies, researchers found that 21%^24^ to 45%^7^ of individuals with cognitive disabilities experienced live discharge compared to 6% to 15% for the general hospice population. These data demonstrate the significant increased risk of live discharge that this population experiences and the importance of future research aimed at reducing this risk.

Researchers have found that geographic location (i.e., distance to and concentration of healthcare facilities) in the US is associated with access to care (i.e. hospice facilities, nurse visitations, and hospitals) as well as variation in population races/ethnicities, and socioeconomic status.^36^ This finding is vital for researchers to understand, as for-profit hospice ownership rose from one-third in 2000 to over two-thirds by 2020.^37^ Many hospice acquisitions have been funded through private equity.^37^ In 2021, 14% of Medicare hospice patients received care from private equity-owned hospices.^38^ Growth in for-profit hospice ownership has been substantial; however, these agencies offer narrower range of services,^39,40^ provide fewer community benefits (i.e. charity care),^39,40^ care for more lower acuity patients,^41^ have higher rates of hospital and emergency department use,^42^ and receive more deficiency citations.^22,39–45^ This systematic review adds to the body of evidence demonstrating significant differences in clinical outcomes by hospice ownership (i.e., increased risk of live hospice discharge for patients with cognitive disabilities serviced by for-profit hospice agencies), identifying three studies that showed these associations.^13,22,35^ More research is also warranted to explore differences in organizational factors, especially private equity ownership and live discharge from hospice.

None of the included studies address if individuals with cognitive disabilities enter hospice with lower mortality risk, so it is not possible to conclude if this contributes to the increased rate of live discharge among this population. However, a study that looked at hospice re-enrollment after live discharge found no significant association between diagnosis, functional score at admission or change between admission and discharge, and mortality within six months after live hospice discharge, suggesting that differences in live discharge may not be related to functional status or mortality risk.^46^ Further exploration on the factors that place individuals with cognitive disabilities at increased risk of live hospice discharge is therefore warranted.

Literature on hospice re-enrollment following live discharge is limited, however a single retrospective study conducted by LeSage and peers (2014) found that only 31% of general population patients who were discharged alive from hospice re-enrolled.^47^ These authors did not include ADRDs (or other cognitive disabilities) as a primary hospice diagnosis in their analyses, therefore conclusions related to the cognitive disability population cannot be drawn. However, it is worth noting that the authors found that 40% of patients died within 6 months of live discharge, suggesting that these patients actually had ongoing hospice eligibility at the time of their deaths.^47^ Further research is warranted to examine if disparities exist in hospice reenrollment following live discharge for patients with cognitive disabilities.

This review identified additional gaps and future possible research opportunities. There were no interventional studies found in our literature search, and all the studies of patients with cognitive disability focused on patients with ADRDs. Given their vulnerability to poor end-oflife outcomes, patients with cognitive disability (e.g., more broadly speaking, specifically in the context of intellectual and developmental disabilities) should be included in future work. As investigators learn more about disparate hospice care among this group, research should move towards intervention development to minimize suboptimal hospice care/end-of-life outcomes for those with cognitive disabilities. Of note, none of the studies in this review followed patients for prolonged periods of time after live discharge. It is vital that researchers explore what happens to patients with cognitive disabilities after live discharge to determine what meaningful interventions this population wants and needs regarding hospice care.

To summarize, the population of individuals with cognitive disabilities is increasing the US, and as they age, there will be subsequent growth in the number of those accessing hospice care. Individuals with cognitive disability experience disparate access and care quality in many aspects of their medical care,^21,48–51^ however data on their hospice use is limited. This review summarized the evidence to date and demonstrated a need for enhanced understanding of outcomes and potential hospice care disparities among this population.^10,12,21^ While we sought to find research that included patients with a variety of cognitive disabilities, this aim proved to be more difficult than anticipated. The vast majority of the data focused on individuals with ADRDs. In the future, it would be beneficial to have data on individuals with other forms of cognitive disabilities, such as intellectual and developmental disabilities and acquired brain injuries as these populations are at increased risk of adverse end-of-life outcomes. Investigators conducting hospice research should also consider assessing in-hospital death, where many acquired brain injuries (i.e., traumatic brain injury and stroke) deaths occur. Additionally, future research should include work targeting individuals with intellectual and developmental disabilities as this group was not readily identified in studies included in our review. Our hope is in the future to involve individuals directly impacted by this work as stakeholders and collaborators, because as the saying goes, “nothing about us, without us.”^52^

## Limitations

While we conducted a systematic search, we may have missed some published articles. We had justification for excluding studies prior to 2014 but may have excluded data relevant to our research question. We restricted our search to deaths outside of the hospital, which may have failed to capture studies of individuals with cognitive disability who died in the hospital following hospice care. Our definition of cognitive disabilities was intended to be comprehensive but was developed by our research team and could have resulted in exclusion of other studies of relevant populations. Further, our inclusion of only adult patients limits our evidence synthesis given that a large subset of patients with intellectual and developmental disabilities are pediatric patients who potentially qualify for/access hospice care. All the studies we identified focused on individuals with ADRDs, thus generalizability to the larger cognitive disability community is limited. Furthermore, our search was limited to the US, which limits generalizability to other countries.

## Conclusions and Implications

Hospice disparities, including live discharge from hospice, are a fact for individuals with cognitive disabilities, one such example is live discharge from hospice. As the population of individuals with cognitive disabilities in the US continues to grow, it is vital that researchers explore the underlying etiology of live discharges, to best understand the problem, and to find solutions to address it. In reducing live discharges from hospice for individuals with cognitive disabilities we can ensure equitable end-of-life care for vulnerable populations, reduce healthcare spending, and prevent patient and caregiver duress.

## Supplementary Material

1

## Figures and Tables

**Table 1.** Study Characteristics for Included Studies

Author, year	Study Aim(s)	Data Source	Design and Statistical Approach	Sample, Setting, and Study Period
Aldridge et al, 2022	Examine differences in hospice use patterns between those with comorbid dementia and those with a primary hospice diagnosis of dementia	Health and Retirement Study linked with Medicare claims	Retrospective cohort Bivariate analyses via chi-square and t-tests Multivariable logistic regression to estimate adjusted odds ratios between dementia status and each outcome Assessed potential interaction effects of specific primary hospice diagnoses (cancer and heart failure) and comorbid dementia, and also dementia and low socioeconomic status	Individuals who participated in the HRS study (i.e., US adults aged 51+ years), died between 2004 and 2016, and received hospice care. National Sample (N=3123): Female 58.6% Nursing Home Resident 43.2% Race: White 82.5% Black 11.5% Hispanic 6.0%
Hunt et al, 2022	Assess the frequency of provider-initiated (extended prognosis) and patient-initiated (revocation) disenrollment, examine hospice provider and regional variation in disenrollment, and identify individual patient, hospice provider, and regional predictors of disenrollment	Medicare data linked to databases of hospices and regional characteristics	Retrospective cohort Multilevel mixed-effects logistic regression models to estimate a medians odds ratio (MOR) of disenrollment for hospices and regions for each type of disenrollment Used an algorithm based on hospice claims to identify the reason for disenrollment. The choice of a 1-year cutoff was based on several considerations. While Medicare hospice eligibility requires a prognosis of less than 6-months, beneficiaries are not automatically disenrolled at the end of 6-month period and many enrollees—especially those with dementia— are disenrolled after 6-months	Individuals who had their first admission to hospice between July 1, 2012, and December 31, 2016, and were admitted to hospice for a diagnosis of dementia National Sample (N=867695): Hospices 4614 Female 65% Race: White 85.3% Black 7.1% Hispanic 5.2% Other 2.4%
Russell et al, 2017	To report frequencies and associated risk factors for 4 distinct causes of live discharge from hospice	Electronic Medical Records from a single hospice agency in New York City	Retrospective cohort Multinominal logistic regression analyses. Means and percentages were used to describe the study population. Variables were entered simultaneously into a multinomial logistic regression model that was used to examine associations of socio-demographic, clinical, and functional characteristics with live discharge reasons. Separate logistic regression models were used to examine residuals and goodness of fit.	Individuals served by an urban, non-profit hospice organization in New York City between 2013 and 2015 Single City Sample (N=9190): Female 58% Race: White 52.5% Hispanic 20.2% Black 16.1% Asian or other 11.2%
Hunt et al, 2023	The objective was to characterize the association between hospice quality and racial disparities in disenrollment among people with dementia. Specifically, we sought to assess disparities in disenrollment between low- and high-quality hospices, as well as within hospices with similar quality	Medicare hospice fee-for-service claims, the Medicare Beneficiary Summary File, Hospice Provider of Service File, Hospice Public Use File, Neighborhood Atlas, and publicly reported data from the Consumer Assessment of Healthcare Providers and Systems	Retrospective cohort Descriptive Statistics: Summarized characteristics of hospice enrollees and agencies (means, medians, frequencies, percentages). Comparative Analysis: ANOVA: Compared continuous variables across hospice quality and race categories. Chi-Square Tests: Compared categorical variables across hospice quality and race categories. Multilevel Mixed-Effects Logistic Regression: Modeling Racial Differences: Estimated racial differences in disenrollment using a model accounting for fixed effects (race, hospice quality) and random effects (hospice providers). Null Model: Included fixed effects of hospice quality and race, their interaction, and random intercepts for hospice providers. Fully Adjusted Model: Adjusted for patient case-mix and hospice organizational characteristics. Post Hoc Analyses: Predicted Probabilities: Estimated probabilities of disenrollment by race across hospice quality categories for both null and fully adjusted models. Adjusted Odds Ratios (AORs): Between-Hospice Disparities: Calculated AORs comparing disenrollment odds between different hospice quality categories by race. Within-Hospice Disparities: Calculated AORs comparing disenrollment odds within the same hospice quality category by race, adjusting for case-mix and organizational characteristics.	Individuals enrolled in Medicare and diagnosed with dementia, enrolled in hospice between July 2012, and December 2015, and were admitted to hospice with a diagnosis of dementia National Sample (N=673102): Hospices 4371 Non-profit 24.3% For-profit 57.7% Other 18% Female 66% Race: White 85% Black 7.3% Hispanic 6.3% Asian American and Pacific Islander 1.6%
Gianattasio et al, 2023	Describe trends in live discharges and patient length of stay in relation to the 2014 IMPACT Act and the 2016 payment change using Medicare hospice claims from 2008–2019.	The Medicare limited dataset, the Medicare Beneficiary Summary File, and provider-of-service files	Retrospective cohort Linear function pre and post 2014 IMPACT act policy change Modeled trends in live discharge and LOS before (up to May 2013, the “pre-policies period”), during (June 2013 December 2015), and after (January 2016 onward, the “post-policies period”) the set of three policy changes. Specially, we modeled continuous trends in a pre-policies and post-policies periods using linear functions, but provide month-by-month characterization of changes to our outcomes during the June 2013 December 2015 period using monthly indicators Conducted subset analyses using up to the first four ICD codes to identify ADRD and lung cancer patients. We then repeated our analyses excluding government-owned hospices and hospices newly entering or exiting the market during the analytical period	Individuals enrolled in Medicare and hospice between July 2008 and September 2019. National Sample (N=10539912): Female 58.2% Race: White 87.7% Black 7.9% Hispanic 1.7% Other 2.7%
De Vleminck et al, 2018	To characterize the hospices that serve patients with dementia, to compare patterns of hospice disenrollment for patients with dementia and without dementia, and to evaluate patient-level and hospice-level characteristics associated with hospice disenrollment	National Hospice Survey data linked with Medicare claims	Retrospective Cohort Summarized the characteristics of our sample by patient demographic and clinical characteristics and hospice characteristics. Compared the prevalence of patient and hospice characteristics as well as the patterns of hospice disenrollment for the dementia and nondementia group using χ^2^ tests. To characterize the hospices that serve patients with dementia, examined the associations between hospice-level characteristics and the percentage of any patients with dementia they serve (both patients with a primary diagnosis of dementia and patients with a coexisting diagnosis of dementia), using generalized linear models. Estimated separate multivariable models for the 3 patterns of hospice disenrollment. For this, multivariable logistic regression was used to examine the associations between patient and hospice characteristics and the 3 patterns of disenrollment, compared with patients who remained with hospice until death. All tests were performed using techniques to account for the clustering of patient observations within hospices. A 2-tailed P < .05 was used to define statistical significance.	Individuals with Medicare enrolled in hospice between 2008 and 2011. National Sample (N=149814): Hospices 577 For-profit 38% Non-profit 62% Female 71.6% Race White 86.6% Other 13.4%
Luth et al, 2020	Identify demographic, health, and hospice service factors associated with live discharge due to condition stabilization or failure to decline among hospice patients with dementia	Electronic Medical Records from a single non-profit hospice agency in New York City	Retrospective cohort Descriptive statistics for the analytic sample were calculated. Used logistic regression models to identify risk factors for live discharge from hospice (versus death). Tested whether nonlinear terms for hospice delivery (length of service, number of nurse visits, timing of nurse visits) resulted in significantly improved model fit using likelihood ratio tests, McFadden’s R2, and Bayesian measures (AIC and BIC). Predicted probabilities for key findings were graphed.	Individuals 65+ years of age, with dementia, enrolled in hospice with a single agency in New York City between 2013and 2017. Single City Sample (N=2629): Female 72% Race White 55% Black 16% Hispanic 20% Other 9%
Luth et al, 2021	examine the relationship between 1) care location (home vs. nursing home) 2) hospice nurse visit frequency and risk of death within 6 months among hospice patients with dementia	Electronic Medical Records from a single non-profit hospice agency in New York City	Retrospective cohort Descriptive statistics comparing patients who died within 6 months of hospice enrollment to those with a long LOS or live discharge Survival analysis to examine the influence of care setting and nurse visit frequency (Model 1) and the interaction between the two (Model 2) on risk (hazard) of expected death vs. live discharge or long length of stay Cox proportional hazard model with a time-varying effect for nurse visits to identify risk factors for expected death	Individuals 65+ years of age with dementia, enrolled in hospice with a single agency in New York City between 2013 and 2017. Single City Sample (N=3837): Female 72.2% Race White 54.3% Black 15.6% Hispanic 21% Other 9.2%

**Table 2.** Factors Associated with Live Hospice Discharge

	Aldridge et al, 2022	Hunt et al, 2022	Russell et al, 2017	Hunt et al, 2023	Gianattasio et al, 2023	De Vleminck et al, 2018	Luth et al, 2020	Luth et al, 2021
Cognitive Disability	X	X	X	X	X	X	X	X
Risk of Live Discharge for Individuals with Cognitive Disabilities	↑	↑	↑	↑	↓ since IMPACT Act	↑	↑	↑
*Patient-level factor*								
ADRDs Diagnosis	X		X		X	X	X	
Minoritized race or ethnicity	X	X	X	X			X	
Female sex	X	X	X			X		
*Hospice-level factor*								
Home Hospice							X	X
Longer time between RN visits							X	X
Lower Quartile of Quality Ratings		X		X		X		
For-profit Hospice		X		X		X		
