# Supplementary material for: Live Hospice Discharge of Individuals with Cognitive Disabilities: A Systematic Review
Source: J Am Med Dir Assoc. Author manuscript; Available in PMC 2026 Jun 1. (PMC12162226; doi:10.1016/j.jamda.2025.105578)
Supplement: 1 [file NIHMS2074141-supplement-1.docx]

**Supplemental Materials**

Search Queries by Database

| Database | Search Query |
| --- | --- |
| PubMed.gov | (“Hospices" [mesh] OR "hospice care" [Mesh] OR hospice*) AND ("live discharge" OR "live hospice discharge" OR "discharged alive" AND (alzheimer* [Mesh] OR "Dementia" [Mesh] OR dementia* OR "Cognitive Dysfunction* [mesh]”) |
| CINAHL | ((MH Hospices+) OR (MH "hospice care+") OR hospice* ) AND ("live discharge" OR "live hospice discharge" OR "discharged alive" AND ((MH alzheimer*+) OR (MH Dementia+) OR dementia* OR "Cognitive Dysfunction* [mesh]" )) |
| Web of Science | (Hospices OR "hospice care" OR hospice* ) AND ("live discharge" OR "live hospice discharge" OR "discharged alive" AND (alzheimer* OR Dementia OR dementia* OR "Cognitive Dysfunction* [mesh]" )) |

**Supplemental Table 1. Joanna Briggs Institute Checklist**

| Joanna Briggs Institute Checklist Element | Gianattasio 2023 | De Vleminck 2018 | Russell  2017 | Luth  2020 | Luth  2021 | Aldridge  2022 | Hunt  2023 | Hunt  2022 |
| --- | --- | --- | --- | --- | --- | --- | --- | --- |
| *Analytic Cross-Sectional Studies* |  |  |  |  |  |  |  |  |
| Were the criteria for inclusion in the sample clearly defined? | Y | Y | Y | Y | Y | Y | Y | Y |
| Were the study subjects and setting described in detail? | Y | Y | Y | Y | Y | Y | Y | Y |
| Was the exposure measured in a valid and reliable way? | Y | Y | Y | *U* | Y | *U* | Y | Y |
| Were objective, standard criteria used for measurement of the condition? | Y | Y | Y | *U* | Y | *U* | Y | Y |
| Were confounding factors identified? | Y | Y | Y | Y | Y | Y | Y | Y |
| Were strategies to deal with confounding factors stated? | Y | Y | Y | Y | Y | Y | Y | Y |
| Were the outcomes measured in a valid and reliable way? | Y | Y | Y | Y | Y | Y | Y | Y |
| Was appropriate statistical analysis used? | Y | Y | Y | Y | Y | Y | Y | Y |
| Total | **8/8**  **100%** | **8/8**  **100%** | **8/8**  **100%** | **6/8**  **75%** | **8/8**  **100%** | **6/8**  **75%** | **8/8**  **100%** | **8/8**  **100%** |

*Note*. Y Yes; **N** No; *U* Unclear; N/A Not Applicable

**Supplemental Table 3. Key Findings**

| Author, Publication date | Key Findings |
| --- | --- |
| Aldridge et al, 2022 | Comorbid dementia was associated with increased odds of hospice enrollment greater than six months (adjusted odds ratio [AOR] = 1.52, 95% confidence interval [CI]: 1.11–2.09) and hospice disenrollment following six months of hospice (AOR = 2.55, 95% CI: 1.43–4.553).  Having a primary diagnosis of dementia was associated with increased odds of hospice enrollment greater than six months (AOR = 2.62, 95% CI: 1.86–3.68), hospice disenrollment (AOR = 1.82, 95% CI: 1.32–2.51), and hospice disenrollment following six months of hospice (AOR = 4.31, 95% CI: 2.37–7.82). |
| Hunt et al, 2022 | Among 867,695 hospice enrollees with dementia, 70,945 (8.2%) were disenrolled due to extended prognosis and 43,133 (5.0%) revoked within 1-year of their index admission. Of those disenrolled, 70,594 (50.3%) were due to extended prognosis and 43,133 (30.6%) revoked, and the remainder were disenrolled for another reason. The median length of stay was 169 days (IQR 86, 239) for disenrolled due to extended prognosis and 67 days (IQR 19, 157) for revocation.  There was substantial variation in hospice provider disenrollment due to extended prognosis (10th–90th percentile 4.5%−14.6%, adjusted median odds ratio (MOR) 1.89, 95% confidence interval (CI) 1.84, 1.93) and revocation (10th–90th percentile 2.5%−10.1%, MOR 2.12, 95% CI 2.06, 2.17).  Characteristics associated with higher odds of disenrollment due to extended prognosis include younger age (AOR 2.29, 95% CI 2.17, 2.41), female sex (AOR 1.51, 95% CI 1.48, 1.54), minoritized race and ethnicity (AOR 1.24, 95% CI 1.20, 1.28 for Black PWD), Medicaid dual-eligible (AOR 1.28, 95% CI 1.25, 1.30), Medicare Part C enrollee (AOR 1.03, 95% CI 1.01, 1.05), residing at home (AOR 1.62, 95% CI 1.58, 1.66) or assisted living (AOR 1.62, 95% CI 1.58, 1.66) versus a nursing home. |
| Russell et al, 2017 | Roughly one in five hospice patients were discharged alive (21%; n = 1911). Acute hospitalization was the most frequent reason for live discharge (42% of all live discharges; n = 802). Additional reasons included elective revocation to resume disease-directed treatments (18%; n = 343), disqualification (14%; n = 271), and service transfers or moves (26%; n = 495).  Risk for acute hospitalization was higher among younger patients (age AOR = 0.98 [95% CI = 0.98–0.99] P < .01), racial/ethnic minorities (Hispanic AOR = 2.23 [CI = 1.82–2.73] P < .001; African American OR = 2.46 [CI = 2.00–3.03] P < .001; Asian/ other OR = 1.63 [CI = 1.25–2.11] P < .001), and patients without advance directives (AOR = 1.41 [95% CI = 0.98– 0.99] P < .001).  Disqualification occurred much more frequently among patients with non-cancer diagnoses, including dementia (AOR = 13.14 [95% CI = 7.96–21.61] P < .001) and pulmonary disease (AOR = 11.68 [95% CI = 6.58–20.74] P < .001). Transfers and service moves were more common among Hispanics (AOR = 1.56 [95% CI = 1.45–2.34] P < .001), African Americans (AOR = 1.35 [95% CI = 1.03–1.79] P < .05), patients without a primary caregiver (AOR = 1.35 [95% CI = 1.09–1.67] P < .001), and those without advance directives (AOR = 1.30 [95% CI = 1.07–1.58] P < .01). |
| Hunt et al, 2023 | Likelihood of disenrollment was higher in hospices in the lowest quartile of quality ratings (vs. highest quartile) for both White (adjusted odds ratio [AOR] 1.12 [95% confidence interval 1.06–1.19]) and minoritized PWD (AOR range 1.2–1.3) and was substantially higher in unrated hospices (AOR range 1.8–2.0).  Within both low- and high-quality hospices, minoritized PWD were more likely to be disenrolled compared with White PWD (AOR range 1.18–1.45). |
| Gianattasio et al, 2023 | Live discharge rates were consistently higher in for-profit hospices than in nonprofit/government-owned hospices.  Prepolicies vs. postpolicies differences in monthly discharge trends were also more pronounced in for-profit hospices. In for-profit hospices, overall rates of decline slowed from -0.16 percentage points per month (95% CI: -0.17, -0.15) during the prepolicies period to no significant change (0.01 percentage points per month, 95% CI: 0, 0.02) during the postpolicies period. Conversely, in nonprofit/government-owned hospices, overall rates slowed from -0.10 (95% CI: -0.11, -0.08) to -0.04 (95% CI: -0.05, -0.02) percentage points per month. |
| De Vleminck, et al, 2018 | 7328 patients (4.9%) had a primary diagnosis of dementia. Hospices caring for patients with dementia were more likely to be for-profit, larger sized, provide care for more than 5 years and serve a large (>30%) percentage of nursing home patients. Patients with  dementia were less likely to disenroll from hospice in conjunction with an acute hospitalization or emergency department visit and more likely to disenroll from hospice after long enrollment periods (more than 165 days) as compared with patients without dementia.  No significant difference was found between patients with and without dementia for disenrollment after shorter enrollment periods (less than 165 days). In the multivariable analyses, patients were more likely to be disenrolled after 165 days if they were served by smaller hospices and hospices that served a small percentage of nursing home patients. |
| Luth et al, 2020 | White hospice patients with dementia, African American and Hispanic hospice patients with dementia experienced increased risk of live discharge (African American: AOR: 2.42, 95% CI: 1.34–4.38; Hispanic: AOR: 2.99, 95% CI: 1.81–4.94). Home hospice (AOR: 7.57, 95% CI: 4.04–14.18), longer length of service (AOR: 1.04, 95% CI: 1.04–1.05), and more days between nurse visits and discharge (AOR: 1.86, 95% CI: 1.56–2.21) were also associated with live discharge. |
| Luth et al, 2021 | Thirty-nine percent (39%) of patients experienced live discharge or long length of stay.  Home hospice patients were more likely to experience live discharge or long length of stays (HR for death: 0.77, 95%CI: 0.69–0.86, p < 0.001).  Frequency of nurse visits was inversely associated with live discharge and long lengths of stay (HR for death: 2.87, 95%CI: 2.47–3.33, p < 0.001). |

**Supplemental Materials**

*Excluded Studies and Justifications*

Wrong Study Design

1. Wladkowski SP. Live Discharge from Hospice and the Grief Experience of Dementia Caregivers. *J Soc Work End Life Palliat Care*. 2016;12(1-2):47-62. doi:10.1080/15524256.2016.1156600
2. Wladkowski SP. Dementia Caregivers and Live Discharge from Hospice: What Happens When Hospice Leaves?. *J Gerontol Soc Work*. 2017;60(2):138-154. doi:10.1080/01634372.2016.1272075
3. Zhang Y, Luth EA, Phongtankuel V, Ling W, Zhang M, Shao H. Factors associated with preventable hospitalizations after hospice live discharge among Medicare patients with Alzheimer's disease and related dementias. *J Am Geriatr Soc*. 2023;71(11):3631-3635. doi:10.1111/jgs.18505
4. Stephanie P Wladkowski, Cara L Wallace, Karla Washington, Live Discharge from Hospice with Dementia: Challenges to Supporting the Patient-Caregiver Dyad, *Innovation in Aging*, Volume 6, Issue Supplement_1, November 2022, Page 685. doi.org/10.1093/geroni/igac059.2514
5. Wladkowski SP, Wallace CL. The Forgotten and Misdiagnosed Care Transition: Live Discharge from Hospice Care. *Gerontol Geriatr Med*. 2022;8:23337214221109984. Published 2022 Jul 12. doi:10.1177/23337214221109984
6. Dougherty M, Harris PS, Teno J, et al. Hospice Care in Assisted Living Facilities Versus at Home: Results of a Multisite Cohort Study. *J Am Geriatr Soc*. 2015;63(6):1153-1157. doi:10.1111/jgs.13429
7. Wladkowski SP, Wallace CL. Live discharge from hospice care: psychosocial challenges and opportunities. *Soc Work Health Care*. 2020;59(7):445-459. doi:10.1080/00981389.2020.1784356
8. Wladkowski SP. Exploring the Experiences of Live Discharge from Hospice for Dementia Caregivers. *The Gerontologist.* 2015;55(Suppl_2):805. doi.org/10.1093/geront/gnv437.04
9. Wladkowski SP, Wallace CL. Current Practices of Live Discharge from Hospice: Social Work Perspectives. *Health Soc Work*. 2019;44(1):30-38. doi:10.1093/hsw/hly040
10. Wladkowski SP, Wallace CL, Gibson A. A Theoretical Exploration of Live Discharge from Hospice for Caregivers of Adults with Dementia. *J Soc Work End Life Palliat Care*. 2020;16(2):133-150. doi:10.1080/15524256.2020.1745351
11. Hunt LJ, Harrison KL. Live discharge from hospice for people living with dementia isn't "graduating"-It's getting expelled. *J Am Geriatr Soc*. 2021;69(6):1457-1460. doi:10.1111/jgs.17107
12. Wladkowski SP, Enguídanos S. Alzheimer's Disease and Related Dementias: Caregiver Perspectives on Hospice Re-Enrollment Following a Hospice Live Discharge. *J Palliat Med*. 2023;26(10):1374-1379. doi:10.1089/jpm.2023.0059
13. Dolin R, Hanson LC, Rosenblum SF, Stearns SC, Holmes GM, Silberman P. Factors Driving Live Discharge From Hospice: Provider Perspectives. *J Pain Symptom Manage*. 2017;53(6):1050-1056. doi:10.1016/j.jpainsymman.2017.02.004
14. Campbell RW. Being discharged from hospice alive: the lived experience of patients and families. *J Palliat Med*. 2015;18(6):495-499. doi:10.1089/jpm.2014.0228

Wrong Outcome

1. Wang SY, Aldridge MD, Gross CP, Canavan M, Cherlin E, Bradley E. End-of-Life Care Transition Patterns of Medicare Beneficiaries. *J Am Geriatr Soc*. 2017;65(7):1406-1413. doi:10.1111/jgs.14891
2. Unroe KT, Sachs GA, Hickman SE, Stump TE, Tu W, Callahan CM. Hospice use among nursing home patients. *J Am Med Dir Assoc*. 2013;14(4):254-259. doi:10.1016/j.jamda.2012.10.006
3. Lin PJ, Zhu Y, Olchanski N, et al. Racial and Ethnic Differences in Hospice Use and Hospitalizations at End-of-Life Among Medicare Beneficiaries With Dementia. *JAMA Netw Open*. 2022;5(6):e2216260. Published 2022 Jun 1. doi:10.1001/jamanetworkopen.2022.16260
4. Harrison KL, Cenzer I, Ankuda CK, Hunt LJ, Aldridge MD. Hospice Improves Care Quality For Older Adults With Dementia In Their Last Month Of Life. *Health Aff (Millwood)*. 2022;41(6):821-830. doi:10.1377/hlthaff.2021.01985
5. Parast L, Tolpadi AA, Teno J, Elliott MN, Price RA. Variation in Hospice Experiences by Care Setting for Patients With Dementia. *J Am Med Dir Assoc*. 2022;23(9):1480-1485.e6. doi:10.1016/j.jamda.2022.03.010

Wrong Population

1. Wang SY, Aldridge MD, Canavan M, Cherlin E, Bradley E. Continuous Home Care Reduces Hospice Disenrollment and Hospitalization After Hospice Enrollment. *J Pain Symptom Manage*. 2016;52(6):813-821. doi:10.1016/j.jpainsymman.2016.05.031
2. Wladkowski SP, Wallace CL, Coccia K, Hyde RC, Hinyard L, Washington KT. Live Discharge of Hospice Patients with Alzheimer's Disease and Related Dementias: A Systematic Review. *Am J Hosp Palliat Care*. 2024;41(2):228-239. doi:10.1177/10499091231168401

Deemed Poor Quality

1. Clayton MF, Utz R, Iacob E, et al. Live hospice discharge: Experiences of families, and hospice staff. *Patient Educ Couns*. 2021;104(8):2054-2059. doi:10.1016/j.pec.2021.01.002
